# What kind of innovation state matters for social justice? Learning from Poulantzas and going beyond

**DOI:** 10.1007/s43253-023-00099-6

**Published:** 2023-06-12

**Authors:** Theo Papaioannou

**Affiliations:** 1 Development Policy and Practice, The Open University, Gardiner 1 Building Walton Hall, Milton Keynes, MK7 6AA UK; 2 Innogen Institute and Development Policy & Practice, The Open University, Gardiner 1 Building – Room 106, Walton Hall, Milton Keynes, MK7 6AA UK

**Keywords:** Innovation state, Social justice, Political economy, Capitalism, Socialism, B30, P16

## Abstract

In the twenty-first century, the notion of the state and its role in innovation and development have become dominant topics of theoretical and empirical inquiry. Although contemporary innovation theorists clearly unpack the myth of market fundamentalism in industrial policy and practice of neo-liberal states, they do not seem to explain precisely how come such states have been justified to play extensive roles in the economy. This paper provides a theoretical explanation by drawing lessons from Poulantzas’ approach to the state and going beyond it to consider alternatives. Accordingly, it conceives the innovation state as a result of the social division of labour and as a condensation of conflicting social relations which have their own materiality. The paper argues that whatever form the innovation state has taken in the western world since the industrial revolution, this has remained predominantly capitalist. Thus, it reproduces the social division of labour that is exploitative and unjust, delivering most benefits of innovation to dominant classes and excluding the very poor and the marginalised. The kind of innovation state that matters for social justice is a non-capitalist one, promoting pluralism of societies of equals through innovation.

## Introduction

In the twenty-first century, the notion of the state and its role in innovation and development have become dominant topics of theoretical and empirical inquiry. The state is generally defined as ‘… a single, unified source of political authority for a territory, drawing upon the undivided loyalties of its population, operating in a well-organised and permanent way, and directed towards the interests of the whole society’ (Dryzek and Dunleavy 2009: 2). Yet, this general definition is problematic because it overlooks the historical fact that the state is neither a unitary subject nor a smooth apparatus of political authority. Instead, as Poulantzas (1978) insisted, the state is an institutional ensemble which mobilises consent vis-à-vis the dominated classes in society and organises the hegemony within the power bloc of dominating classes. This institutional ensemble of the state suffers from internal contradictions and conflicts. Certainly, as Skinner (1989) has shown, the character of the state is impersonal. This means that it is distinct from its rulers but also from the ruled. Features of the state include governing institutions, collectively binding decision-making, monopoly of legitimate use of force, and sovereignty and clear distinction between public and private realms of activity (ibid).

In contrast to the state, innovation is defined as a source of novelty and technological change. According to Freeman (1991), innovation is about the capacity of people to exploit a new idea or method for producing material and social benefits. In his view, exploitation of new ideas and methods is a synonym to commercialisation. In fact, the benefits of innovation are not only distributed through the market but also through non-market institutions, including health and education systems. In this sense, innovation can involve the development of new products and processes, including technologies and services (Smith and Stirling 2018). Features of innovation include processes of knowledge generation through scientific research, knowledge transfer through interactive learning, collective effort for product development and value creation, management of innovation processes, and commercialisation of inventions.

Theorists such as Block (2008), Block and Keller (2011), Mazzucato (2014), and many others have argued that the state has been crucial for promoting innovation and technological change. Even within neo-liberal institutional settings like those of the USA and the UK, the state has managed to perform a hidden developmental role that delivered investments in innovative technologies, including the Internet and the iPhone. Although innovation theorists clearly unpack the myth of market fundamentalism^1^ in industrial policy and practice of neo-liberal states, they do not fully explain how come such states have been justified to play extensive roles in economy and society. More importantly, they do not admit the fact that these hidden developmental states still reproduce the capitalist social division of labour that is exploitative, delivering most benefits to a power bloc of dominant classes, including bourgeoisie classes, and leaving powerless classes behind, including working classes. The values and positions of a power bloc (that represents the unity of several fractions under the leadership of one dominant fraction that claims to promote the same interests as capital) tend to be privileged by research and development (R&D) driven innovations over other less powerful classes including working classes. Therefore, democracy is limited (Smith and Stirling 2018) and social justice cannot be achieved in the diffusion of new technologies.

The question that arises is, in essence, what kind of innovation state matters? Is the kind of state that currently reproduces unequal and exploitative social relations of innovation via the social division of labour or is the kind of state that promotes social equality and inclusion via a reconciliation of social divisions in production of novel goods and services? To answer this question, there is a need for more in depth and critical theorisation of the state than that of contemporary innovation theorists. Putting forward a plausible argument about the innovation state presupposes proper understanding of the state as such. To satisfy this need, Poulantzas’ theory of the state will be revisited. Unfortunately, this theory is absent from the current theorising of the state by innovation thinkers. This absence is particularly noticeable in political economy journals which are preoccupied with institutional approaches to technical and technological change. This paper insists that contemporary innovation theorists can learn from Poulantzas. Although his theory has been criticised over the years for its structuralist foundations (Miliband 1970; Laclau 1975; Block 1987), its advantage remains twofold: first, it conceives the state as a result of the social division of labour and therefore acknowledges the unity between politics and economics; second, it considers the state to be a condensation of conflicting social relations which have their own materiality and therefore does not abstract from the historical limitations of capitalism. However, despite advantages, Poulantzas’ theory faces its own limitations. The most important of them is that it offers just one alternative to capitalist innovation state, i.e. democratic socialism. Yet, there may be other alternatives too, emerging from the bottom up, especially in the global South. To grasp them, we need to move beyond Poulantzas’s structuralism and towards embracing pluralism and the role of public action in social change.

In what follows, the argument of this paper will be developed in five sections. Section 2 discusses key accounts of the innovation state. Section 3 revisits Poulantzas’ theory of the state. Section 4 attempts to go beyond Poulantzas, arguing the kind of innovation state that matters is the state that emancipates itself from the social division of labour and instead relies on (non-exploitative) social co-operation, producing just outcomes. Section 5 concludes by summarising the main argument of this paper.

## Key accounts of innovation state

The twenty-first century state is (and ought to be) concerned about innovation. This is so for all the reasons which Bush (1945) explained in his infamous *Science*, *the Endless Frontier*, including economic growth, employment, public health, and natural security. For Bush, all the things that innovation helps us achieve are the very objectives of government. However, innovation is not only important for economic growth and employment but also for social justice (Papaioannou 2020; Papaioannou and Srinivas 2019). Of course, social justice as such is a contested concept. As Buchanan et al. (2011: 308) stress, ‘Theorising about justice is notoriously afflicted … with both disagreement and uncertainty. There is disagreement between consequentialists and deodologists, between proponents of ‘positive’ rights and libertarians, between egalitarians, prioritarians, and sufficientarians, and among egalitarians as what the ‘currency’ of egalitarian justice is (well-being, opportunity for well-being, resources or capabilities)’. This paper takes an egalitarian perspective of justice, focusing primarily on social relations instead of resources. From this perspective, innovation can create favourable conditions for achieving social justice or pose risks of injustice, i.e. making some people worse off or exacerbating their disadvantaged positions (Papaioannou 2018; Buchanan et al 2011). Economists and innovation theorists tend to provide rather competing accounts of innovation state. Some of these accounts are explicit while some others are implicit. One might make a rough distinction between five key accounts of innovation state.

The first is the neo-classical account of innovation; it understands the state as a marginal actor in the process of generating new technologies. Only when the market fails to deliver innovation the state intervenes to address the failure. Consciously oversimplifying, neo-classical economists (e.g.Jevons 1888; Walras 2014; and others) were rather sceptical of the role of the state in the market, including regulatory interventions in the process of supply and demand (Freeman and Soete 1997) and redistribution. Their account of innovation state, even though implicit, was static and a-historical. It constituted an intellectual break from the classical tradition of Smith and Marx who emphasised technological drivers of historical change through the social division of labour in production. The state was conceived as a result of that division of labour. Certainly, the division of labour within capitalism leads to increased efficiency and productivity. However, its social nature also leads to unjust relations of exploitation.

The second account of innovation state is that of Schumpeter. He insisted that only ‘new combinations’ which add value to social system can be defined as innovations (Schumpeter 1983). Entrepreneurship is about turning inventions into profitable use (Kaplinsky 2021) and thereby innovations. In contrast to neo-classicals, Schumpeter emphasised the historical and, above all, the evolutionary process of change through which innovation impacts on capitalist economic development. Although for him, the individual entrepreneur is the key economic agent, the state and institutions also play crucial role in the background. Their role consists of maintaining a particular structure of society and organisation of production without which entrepreneurs would not be able to take opportunities and introduce new combinations in large oligopolistic corporations. In his *Capitalism*, *Socialism and Democracy*, Schumpeter (1983) tends to consider the state as part of a wider innovation system. In his introduction to this work, Stiglitz (2010: xiii) points out that ‘Today, we think of the role of government in helping create the most transformative innovations of the twentieth century, including the Internet; but even in the nineteenth century, government financed the telegraph line and not only supported the research that provided foundations of America’s increase in agricultural productivity but provided the extension services that brought that knowledge to farmers’. Schumpeter brings back innovation theory to its classical roots, especially to the theories of Marx and Smith, in the sense that he appreciates the inherent evolutionary nature of capitalism. Therefore, his theory of economic development is underpinned by Smith and Marx’s evolutionary perspective. Clearly, as Rosenberg (2011) points out, Schumpeter and Marx shared the same vision of capitalism as a social system that had its own internal logic and transformation. In this intellectual context, the state is explicitly considered a fundamental category for the understanding of technological development. Clearly, Schumpeter makes different methodological choices from Marx and Smith. In his early writings, he focuses mainly on the role of individual entrepreneurs in technological innovation and social change, keeping economics and sociology apart. In his later works, however, Schumpeter emphasises that in modern corporation, innovation is a collective, routinized process. Generally speaking, his theory of innovation and economic development is in line with classical analysis of capitalism as a social and economic system driven by competition in the market. The latter would be impossible without politics, state policies, and institutions such as private ownership and credit creation in the background to regulate the process of free competition and provide guarantee of economic transactions. In his *Capitalism*, *Socialism and Democracy*, Schumpeter (1976: 94) agrees with Marx that ‘…politics and policies are not independent factors but elements of the social process we are analysing …’. However, for the purpose of his ‘pure’ economic argument, he prefers to consider them as external factors to the world of businesses. Certainly, Schumpeter borrowed large parts of his theory of innovation from the German Historical School, especially Sombart, but also from the Austrian School of Economics, especially Böhm-Bawerk. Given their dispute about objectivist (historical) and subjectivist (theoretical) approaches to economics (Methodenstreit), Schumpeter avoids taking sides and instead tries to think of history and theory as mutually inclusive methods for understanding economic development. Thus, he initially explains innovation through new combinations by individual entrepreneurs (i.e. subjectivism akin to Böhm-Bawerk’s theory) within capitalism that historically evolves through (discontinuous) stages (i.e. historicism akin to Sombart and Marx). However, later, he comes even closer to the German historical school that put great emphasis on both entrepreneurs and the state. These institutions were not seen as mutually exclusive. Instead, the state (as the set of formal rules) was seen as an enabling factor of entrepreneurship and innovation.^2^

The fourth account of innovation state is that of Keynes. He proposed the end of ‘laissez fair’ in 1926 and a few years later argued for government regulation of savings and investment. These proposals, in Keynes’ view, could combat unemployment and deal with the economic crisis of 1929–1930. In his *General Theory of Employment*, *Investment and Money* in 1936, he clearly stressed that his aim was to contrast his arguments with those of classical liberal economists defending the role of the state in economy against the ‘laissez fair’ market (Keynes 1936). In addition, Keynes was one of the first economists who saw his task to provide advice to government and policymakers on issues of public investment. Subsequently, innovation scholars such as Chris Freeman and Richard Nelson followed his example, engaging actively with decision makers (Lundvall 2013). Keynes was critical of financial speculation and casino capitalism. His main interest was in productive investment for employment and growth. As Mazzucato (2018) points out, Keynes insisted on the need for the state to do what is not being done by the market, i.e. invest in and support the generation of risky technological innovations. Keynes was aware of the uncertainty (or risk) of innovation. However, according to him, ‘It is safe to say that enterprise which depends on hopes stretching into future benefits the community as a whole. But individual initiative will only be adequate when reasonable calculation is supplemented and supported by animal spirits, so that the thought of ultimate loss which often overtakes pioneers, as experience undoubtedly tells us and them, is put aside as a healthy man puts aside the expectation of death’ (Keynes 1936: 162).

The fifth account of innovation state is that of Hayek. He directly challenged Keynes’ belief in strong state, offering the alternative idea of the market as a spontaneous order. This type of order is self-organising and arises endogenously through forces which are not deliberately created (Hayek 1973: 36–37). The market as a spontaneous order evolves without state intervention but by following general rules. Therefore, innovation is generated through a complex and evolutionary process of adaptation to these rules which embody tacit knowledge. According to Hayek (1960: 26), ‘Out habits and skills … our tools, and our institutions – all are … adaptations to past experience which have grown up by selective elimination of less suitable conduct’. The human mind let alone the state cannot master this process. This implies that rational interventions in the market through state policies might have negative consequences for this institution and individual interests. Hence, Hayek argues, the market should be left alone to generate innovations spontaneously, often as unintended results of rational individual actions. Given its evolutionary nature, the market will select and retain the strongest possible innovations from a variety of candidates. Therefore, there is no need and/or justification for the state to pick up winners in the evolutionary market (Hayek 1960). The state should remain neutral towards different conceptions of innovation and technological good. The application of liberal notion of innovation defines the Hayekian innovation state.

It might be argued that contemporary innovation theories either combine the Schumpeterian and the Keynesian innovation state (Freeman 1981; Perez 2016, 2002; Nelson 1992; Lundvall 1992, 2007; Block 2008; Block and Keller 2011; Mazzucato 2014; 2018) or they reconstruct the Hayekian evolutionary perspective (Saviotti and Metcalfe 1991; Potts 2018; Witt 2016). The latter appears to underplay the role of political judgements in defining the direction of technological innovation. This task is left to the market spontaneous forces. In contrast, the former perspective seems to accept the importance of politics and the state in defining the direction of innovation (Papaioannou, 2012). Directionality of innovation is crucial for social reproduction in terms of social justice, whether certain technologies, e.g. nuclear power, artificial intelligence (AI), and information and communication technologies (ICT), are politically desirable and beneficial for certain social classes or ethnic minorities depend on the extent to which they include or exclude the needs of these people.

Unfortunately, contemporary innovation theorists seem to overlook the historical fact that, whether Schumpeterian, Keynesian, Hayekian, or even neo-classical, the western innovation state since the first industrial revolution has remained predominantly capitalist. This means that it has been biased towards supporting the interests of power blocs organised by the state, failing to promote a fair system of cooperation where risks and benefits of innovation are distributed in reciprocal way. The capitalist state, especially after the World War II, facilitated innovation as apolitical and conflict-free process (Pfotenhauer and Joakim 2017) that could increase economic growth for the sake of these power blocs. What Schot and Steinmueller (2016) identify as first framing of science and technology corresponds to the post-war capitalist innovation state that focused on research and development (R&D) for defence, telecommunications, medical research, and engineering. As they note (ibid: 4), ‘A broad consensus emerged that the state could and should play an active role in financing scientific research on the premise that scientific discoveries would trickle down to practice through applied R&D by the private sector’. This broad consensus was mainly between different fractions of capital and dominant classes which had to step back from their pre-war market fundamentalism in order to resume capital accumulation. These classes accepted that the capitalist state had to combine policy measures (i.e. construct innovation policy) to influence the conditions of innovation in firms (e.g. encourage the transfer of military and space research to the civil economy).

As Poulantzas (1978) has stressed, dominant classes are very fragmented but tend to establish specific relations of proximity or alliance with the capitalist state through lobbies and networks of power which shape its structures. This is the reason why, in Europe and in the USA, power blocs of dominant classes have encouraged even right-wing governments to play increasing role in underwriting the advance of new technologies and endorsing development policies that support cutting edge research (Block 2008). Such policies have led to commercialisation of innovative products by private firms and hence to further capital accumulation. In addition, they have maintained the standards of living of working classes and sustained low unemployment, marginalising social struggles against capitalism.

Certainly, as Block (2008) correctly observes, the politics of capitalist innovation state in Europe and in the USA have been different from those in other regions, e.g. East Asia in the decades after World War II. The latter policies have been centralised and bureaucratic whereas the former have been decentralised and networked. For this reason, Block (ibid) talks of Developmental Bureaucratic State (DBS) in East Asia and of Developmental Network State (DNS) that is hidden in the USA and visible in Europe. According to him, ‘Instead of the DBS reliance on providing firms with incentives, the DNS is much more ‘hands on’; it involves public sector officials working closely with firms to identify and support the most promising avenues for innovation’ (ibid: 172).

Although other theorists such as Amsden (1989)^3^ and earlier than her Gerschenkron (1962) would almost certainly dispute that DBS in late industrialised countries of East Asia, e.g. Taiwan and Korea, were not ‘hands on’ establishments, they would agree with Block that post-World War II industrialised innovation and development have been indeed state led. In both DBS and DNS, however, one can notice that the political economy of innovation revolves around certain relations of production which remain exploitative and unjust. These social relations are reproduced politically through the capitalist state and not through a mystical process of social evolution. Again, as Poulantzas (1978: 17) pointed out, ‘The political field of the state (as well as the sphere of ideology) has always, in different forms, been present in the constitution and reproduction of the relations of production’. Neo-Schumpeterian theories of innovation (Mazzucato 2014, 2021) propose to remake capitalism just by strengthening DNS to become riskier and more entrepreneurial in terms of promoting socially desirable productive forces (e.g. health technologies, green innovations) but tend to overlook the importance of relations of production (e.g. private property and exploitative relations of capital and labour) This kind of innovation state is bound to fail to achieve inclusion and social equality.

In the next section, I will revisit Poulantza’s theory of the state. This theory puts forward an alternative notion of the state as a social relation that has the potential to organise the transition from capitalism to democratic socialism. I will insist, however, that a democratic socialist innovation state cannot just be the only alternative to capitalist innovation state. There may be other non-capitalist state of innovation alternatives emerging from the bottom up and through public action. Innovation scholars should investigate further these alternatives in both normative and empirical terms. The objective should be to run a feasibility test before arriving at any conclusion about the plausible state of innovation in the twenty-first century.

## Revisiting Poulantzas’ theory of the state

At first glance, the discussion of Poulantzas in this article might seem irrelevant for a number of reasons. First, Poulantzas never used the term ‘innovation state’. Secondly, his references to innovation are in passing and always inside the advanced capitalist states. Poulantzas is interested in the relationship between knowledge and power, particularly the way in which scientists ‘… have become state functionaries …’ (Poulantzas, 2014: 57). Thirdly, he is mainly a theorist of capitalist formations. However, the discussion of Poulantzas is important from an explanatory perspective. In contrast to theorists of innovation state, he is a Marxist thinker who developed a broad political theory of the state drawing on Gramsci’s notion of hegemony, i.e. the exercise of intellectual, moral, and political leadership by the ruling class to achieve the successful reproduction of ‘active consent’ of dominated groups. According to Jessop (1982: 153), ‘Poulantzas is the single most influential Marxist political theorist of the post-war period and, up to his premature death in 1979 he produced a significant body of work on the capitalist state …’. Jessop provided a magisterial survey of Poulantzas’ theoretical development and his notion of the state. This work suggests that Poulantzas elaborated the state as a relation between socio-economic forces. Indeed, in his *State*, *Power*, *and Socialism*, he argued that ‘The (capitalist) State should not be regarded as an intrinsic entity: like ‘capital’ it is rather a relationship of forces, or more precisely the material condensation of such relationship between classes and class fractions, such as this is expressed within the state in a necessarily specific form’ (Poulantzas 1978: 128). This argument reflects Poulantzas’ concern with the class nature of the state and the historical fact that it privileges some agents over others. However, the actualisation of such state biases towards specific agents is relative to the changing balance of forces, strategies, and tactics within the state apparatus (Jessop 2016).

In his reply to Miliband and Laclau’s critiques of his theory, Poulantzas explains that ‘… the term ‘relative’ in the expression ‘relative autonomy’ of the state (relative in relation to what and to whom?) *here* refers to the relationship between state and dominant classes (i.e. relatively autonomous in relation to the dominant classes). In other words, it refers to the class struggle within each social formation and to its corresponding state forms’ (Poulantzas 1976: 72). However, Poulantzas stresses that the degree of autonomy of the state varies across different contexts and historical forms of the state. For example, the DNS in Europe might be thought as more autonomous than the DNS in the USA. The bottom line for Poulantzas is this: ‘… conceiving of the capitalist state as a relation, as being structurally shot through and constituted with and by class contradictions, means firmly grasping the fact that an institution (the state) that is destined to reproduce class divisions cannot be monolithic fissure less bloc, but is itself by virtue of its very structure divided. The various organs and branches of the state (ministries and government offices, executive and parliament, central administration and local and regional authorities, army, judiciary, etc.) reveal major contradictions among themselves …’ (ibid). In essence, these contradictions explain why the state can maintain some relative autonomy from dominant classes when it comes to actual policy making. Non-Marxist theorists such as Skocpol (2008) also recognise so, emphasising the state capacity to take autonomous decisions and design policies which are not always reducible to class interests.

Although Poulantzas is not an innovation state theorist, his explanatory account of the capitalist state has implications for innovation and related public policies. These implications should be taken seriously by contemporary innovation theorists. First, the capitalist state can set up a system of innovation and intervein in the market to support the competitive advantage of domestic firms. Even though illiberal, since the 1980s, the capitalist innovation state has operated within what Schot and Steinmueller (2016: 9) have characterised as ‘Framing 2: National Systems of Innovation’. Indeed, the very concept of the system of innovation became popular among academics and policy makers during that decade, even though, as Codin (2020) observes, it goes back to the 1960s. The reason of this popularity was that the concept of systems of innovation enabled a holistic policy approach to innovation that concerned several ministries and public agencies of the capitalist state. Despite the neo-liberal rhetoric of the 1980s, the capitalist state used its autonomy to construct intervention policies of innovation (e.g. measures for economic environment, taxation, trade). It did so to satisfy the desire of dominant classes for more dynamic capital accumulation. The state in the 2020s might do the same, leading what Schot and Steinmueller (ibid) have defined as ‘Framing 3: Transformative Change’. Within this framing, innovation is politically directed to address challenges such as climate change, reduction of equality, poverty, and pollution.

Second, the relations and contradictions within the capitalist state are the direct result of the social division of labour. The latter ‘… expresses itself in the presence of the political and ideological relations within the production process, which has primacy over the technical division of labour’ (Poulantzas 1978: 35). This implies that the capitalist innovation state embodies oppressive and exploitative relations of production. Such relations are at the core of unjust and exclusive technological innovations. What matters is not so much the technical division of labour that leads to certain technologies (e.g. iPhone, GPS) but the relational context within which such technologies emerge. Often, such context is not just exploitative but also gendered and racialised. Overlooking the importance of this context can give the wrong impression that innovation is a natural activity driven predominantly by technical rationality and has nothing to do with the politics of the capitalist state. Yet, innovation is very much political, depending on the social division of labour that the capitalist state promotes. As Freeman and Soete (1997) show in their third edition of *The Economics of Industrial Innovation*, the generation but also the acceptability of innovative products and processes is very much conditioned by politics.

Contemporary innovation theorists tend to emphasise the external role of the state in R&D investments and in technological missions. For example, in her latest book *Mission Economy: A Moonshot Guide to Changing Capitalism*, Mazzucato (2021) considers the state to be a value creator that can be driven by public purpose to provide directions towards more inclusive and sustainable capitalism. In her view, all the state needs to do is to use its capabilities and competencies to set missions for addressing societal challenges, including the environment, poverty, and inequality. However, what Mazzucato and other innovation theorists often do not seem to realise is that the capitalist state has always had great deal of involvement in creating those challenges through unjust innovation. Historically speaking, capitalism has never operated behind the back of the state. Therefore, the latter would have to be thought as part of the former (hence the term ‘capitalist state’). This implies that the state has never been external to the innovation process as a simple regulator or facilitator. Instead, the state has been part of the innovation process through the social division of labour that is in the core of novel technological products and services.

In contrast to Mazzucato and other contemporary theorists’ rather uncritical approach to the state, Poulantzas’ theory can provide the lenses to view the state in relational terms (just like capital) and therefore to shift research focus on oppressive and exploitative relations and structures which enable innovative technologies. Such a shift, by definition, must take account of the power that is not reducible to the state. In Poulantzas’ theory, power is feature of class practices and not of political structures such as the state. In this sense, power is also rooted in the social division of labour and in exploitation. Thus, for example, the division between intellectual and manual labour defines power relations between those who own knowledge and those who do not; those who sell their manual power (even though manual labour also requires some forms of knowledge) and those who buy it in the market. In the current (neo-liberal) capitalist state, the social division of labour is reflected in skilled-biased technological change. According to Planes-Satorra and Paunov (2017: 7), ‘… opportunities arise for those having adequate capacities to participate in innovation, as new jobs require their skills and new entrepreneurial opportunities are emerging. Prospects for many others worsen: routine middle-skilled tasks are increasingly being automated, while jobs at the lower end of the distribution are seeing increased demand but are associated with low wages and low levels of job security’.

In Poulantzas theory, the state engages in intellectual labour. In this sense, it subordinates working classes and actors which tend to engage in manual labour. These include women, immigrants, ethnic minorities, and residents in deprived areas. As he argues, ‘In all its apparatuses (that is not only in its ideological apparatuses but also in the repressive and economic ones) the state incarnates intellectual labour as separated from manual labour; this becomes evident provided that the two are not conceived according to a naturalist-positivist distinction. And it is within the capitalist state that the organic relationship between intellectual labour and political domination, knowledge and power is realised in the most consummate manner’ (Poulantzas 1978: 55–56). In Poulantzas’ theory, given that the state is not an actor, but a relation characterised by internal conflicts and biases, there is a need for coherent project that can be built on this organic relationship and enable the state to act as a unified political force. In the context of contemporary capitalism, this is the project of science, technology, and innovation. Such a project justifies and unifies different social forces under the institutional umbrella of the capitalist state.

However, science, technology, and innovation are by no means dominant functions of the state (throughout the different stages of capitalist system). In Poulantzas’ theory, the very notion of periodization of capitalism applies at the level of social formation and not at the level of tendencies of the mode of production (Poulantzas 1975). Any social formation is highly complex and cannot be necessarily reduced to the consequences of tendencies of the mode of production. Developments in the economic base do not necessarily directly determine superstructure. Social formations should be reconceptualised in terms of economic, political, and ideological structures. Each of these structures has its own autonomy and it is not the case that the economic structure is always dominant in determining social formations. Poulantzas, following Althusser, distinguishes between determination and dominance. In this theory, the economic structure is not always dominant, albeit determinant. This has implications for the periodization of capitalism. For example, in feudal stage, it is the political structure that dominates social formations. By contrast, in laissez-faire and monopoly stages, it is the economic structure that both determines and dominates social formations. In state monopoly capitalism, it is the state that dominates. In any case, if one accepts Poulantzas’ theory that only social formations can be periodized, one can argue that contemporary capitalism and its state can be clearly distinguished from, say, laissez-faire, monopoly, and state monopoly capitalism. This is because contemporary capitalism and its state are more socially formed through science, technology, and innovation than any other stage of capitalism and its state. In contemporary capitalism, the dominant social mechanisms for controlling production are science, technology, and innovation. The state is there to intervein for the sake of reproducing capitalist social relations (Fine and Harris 1979). In this sense, in contemporary capitalism, the state can be characterised as innovation state without implying that innovation is prioritised as determinant function of the state. Rather, innovation is within an ensemble of distinctive economic, political, and ideological (structural) functions which maintain social cohesion in class-divided societies. From this, it follows that the contemporary normal form of state is related to the intensification of political crisis rather than to economic crises tendencies.^4^

Science, technology, and innovation require intellectual labour. The latter is very much supported by the capitalist state through investments in research and development (R&D) which lead to technological progress and strengthen scientific advantage. The market often fails to generate private funds for R&D investments due to lack incentives and the high risk of new technologies. The capitalist state, on the other hand, has specific expectations regarding scientific outputs: these are required to serve the interests of free market economy and maintain the process of capital accumulation. Although the capitalist state invests public money in the scientific and technological process of knowledge generation, the benefits of this collective effort tend to be privatised through formally articulated ownership relations, e.g. IPRs for the sake of specific power blocs of classes which maintain their privileged power over technological innovation. Thus, as has been argued by many scholars (Papaioannou 2018; Foster and Heeks 2013; Srinivas 2014; Cozzens and Sutz 2012), technological innovation world-wide remains exclusive, failing to meet the pressing needs of working classes and the very poor. Due to social division of labour, the needs of working classes and the needs of the very poor are never taken fully on board in R&D of some high-tech innovations, e.g. AI, gene editing, and gene drives. Some other high-tech innovations, e.g. ICTs and automobiles, have to be modified in order to meet the needs of working classes and the very poor. In fact, all R&D driven innovations embody the properties of social division of labour in their design and development. This means that blocs of dominant class privileges and hierarchies are built into their narratives (Winner 1989).

To make things worse, the assumption that all R&D driven innovations are eventually forces for good and somehow benefits will trickle down to the socially excluded is flawed. R&D driven innovation can be both hierarchical and ‘… lead to destructive creation, benefiting the few at the expense of the many, leading to low quality jobs, and creating more problems than it solves. Many technologies are deeply implicated in a set of persistent environmental problems. They contribute to the current resource-intensive wasteful and fossil fuel paradigm of mass production and mass consumption’ (Schot and Steinmueller 2016: 16). Contemporary innovation theorists have proposed that some of these issues could be addressed through more responsible research and innovation (RRI). However, RRI presupposes that everyone’s interests in innovation are taken on board and there are no social divisions in the process of knowledge generation and exploitation. But is this possible within capitalism?

In fact, within capitalism, as Mazzucato (2014) put it, the critical point is to understand the relation between those who bear the risk and contribute public funds to innovation (through the state) and those who appropriate rewards and capital from innovation through the market. But such an understanding presupposes a clear view of the capitalist state as an apparatus that sustains the social division of labour and strives to reproduce it by privatising the collective efforts for innovation and value creation. Although Mazzucato (2018) has criticised the privatisation of innovation and the individualisation of value creation within capitalism, she seems to overlook the historical fact that it is the capitalist state playing the key role in organising such unequal social relations and related power blocs. According to Poulantzas (1978: 59), ‘The state redraws and reproduces the social division of labour within its own being: it is thus the carbon-copy of the relations between power and knowledge such as they are reproduced within intellectual labour itself. This process takes place in a range stretching from hierarchical centralised and disciplinary relations to those concentrated in the various layers and nodal-points of decision making and execution …’. This Poulantzian understanding of the state leaves no doubt that despite abstract differentiations between its apparatus and the economy or its relative autonomy from dominant classes which secure hegemony, the state is very much interwoven with capitalism. Accumulation of capital through the social division of labour that delivers unjust innovation (i.e. innovations generated in an exploitative context of private ownership) is the primary objective of the capitalist state.

If our understanding so far is correct, then it begs the question of whether the capitalist state can be transformed to prevent appropriation of innovation rewards and extraction of public value by power blocs of dominant classes and provide directions towards technological innovations which promote social justice. Some innovation theorists (Mazzucato 2021, 2014; Block 2018; Kaplinsky 2021; Perez 2016) are optimistic about such state transformation from the top-down with the assistance of new technologies, e.g. ICTs and green technologies. Thus, they envisage a missionary state that can focus its policies on problem-specific challenges and can have the ability to co-ordinate, finance, and direct innovation towards high-value activities. For innovation historians such as Schot and Steinmueller (2016), this is the equivalent to a ‘deep transition’ towards system wide transformations. Such transformations can be translated into public missions. For example, to address the challenge of private appropriation of publicly funded innovations, Mazzucato (2014: 189 suggests the following mission: ‘Where an applied technological breakthrough is directly financed by the government, the government should in return be able to extract royalty from its application. Returns from royalties earned across sectors and technologies, should be paid into a ‘national fund’ which the government can use to fund future innovations’. This mission considers the power and willingness of the state to extract royalty from the application of technological innovation as if it was something straightforward. In fact, it is not. The capitalist state might never insist on innovation royalties earned across sectors and technologies for two reasons: first, there is a risk of compromising legitimacy (Papaioannou, 2021) given that different forms of capitalist state (e.g. authoritarian, liberal, welfare, neo-liberal, or libertarian forms of state) face different legitimacy constraints; second, the relative autonomy of the capitalist state stops where the slowing down of capital accumulation begins to undermine the structures of the economic system. The latter represent the materiality of all state institutions which tend to be imposed on society.

As we have argued elsewhere (ibid), we should not expect any radical reconstruction of society and economy to be initiated and/or organised by the capitalist state (the form of which can be more or less entrepreneurial, depending on its legitimacy constraints). Indeed, the historical lesson that we have learned since the first industrial revolution is that capitalism as such cannot change from the top-down let alone be remade by the capitalist state (that strives to reproduce the unjust division of labour in society). Although the periodisation of capitalism implies different social formations throughout the nineteenth, the twentieth, and the twenty-first centuries, the core of capitalist mode of production remains the same. Evolution within capitalism predominantly concerns the means of production and not the relations of production. Thus, at politics level, what can be remade are some capitalist state policies which stand a chance to challenge free market thinking of neo-liberals and to mitigate the consequences of the free competition. However, such policies would not challenge and/or reshape the core of capitalist mode of production and the exploitative relations which characterise the social division of labour.

It might be speculated that contemporary innovation theorists have either bought the argument that ‘there is no alternative’ to capitalism from the bottom up or they think the radical anti-capitalist argument is an excuse for doing nothing to address the current societal challenges, e.g. poverty, inequality, and environment. Whatever the truth is, contemporary innovation theorists seem to have given up on radical systemic change from the bottom-up. Instead, they seem to have endorsed short-term missions which can unify different fractions of subordinated classes and social forces, maintaining the hegemony of dominant power bloc and legitimising a certain kind of capitalism as socially just. Contemporary innovation theorists do not seem to be concerned much about the fact that capitalism will be reproduced through such missions and unity of illusory interests. The purpose of capital will remain to accumulate profits generated through exploitation, and the purpose of the capitalist state (even though relatively autonomous) will remain to provide a political forum for the capital to justify and legitimise its exploitative relations. To put it another way, systemic change that is in the interest of social equality and environmental sustainability will not be achieved through top-down innovation policies no matter how progressive these might be in terms of promoting social experimentation.

Contemporary innovation theorists such as Mazzucato (2021) and Perez (2016) are right of course to argue that ‘We are now in a crucial moment in history similar to the 1930s, requiring thinking and measures as bold as those of Keynes, Roosevelt and Beveridge as ambitious as the Bretton Woods agreements’ (Perez 2016: 199). However, such measures will not change the nature of social division of labour but simply adjust capitalism into the new reality of challenges to save it from its own fate of collapse. Would a combination of Keynesian and Schumpeterian innovation state matter? If so, for whom? I will try to address these questions in the next section.

## The kind of innovation state that matters: going beyond Poulantzas’ theory

Contemporary innovation theorists tend to promote a combination of the Keynesian and the Schumpeterian accounts of innovation state as a solution to the problem of directionality of new technologies. Their hope is that the Schumpeterian motor of innovation will keep entrepreneurs incentivised to take market opportunities and the Keynesian interventionism will turn public investments into socially valuable technological products and services. This way, what Schot and Steinmueller (2016) have framed as ‘transformative change’ will become a reality for socio-technical systems which will be directed to address social and environmental challenges.

However, as has been already implied, the combined Keynesian–Schumpeterian innovation state tends to rely on the same division of labour that other accounts of capitalist state rely (e.g. neo-classical, Hayekian). This means that its mission to solve the problems we face today will not go far enough to change the social division of labour and thereby eliminate exploitative relations of private ownership, domination, and oppression within innovative production of goods and services (e.g. IPRs). In this sense, a combined Keynesian–Schumpeterian innovation state does not really matter for social justice. Privatisation of knowledge and appropriation of innovation rewards and extraction of value from power blocs of dominant classes will continue under such capitalist innovation state albeit mitigated by some social welfare policies. This is exactly what happened after the World War II through the social redistributive policies of the welfare state in Europe. Such policies mitigated social inequality but never dared to eliminate it by putting a brake into accumulation of capital by classes.

It might be argued that what really matters for social justice is to build an innovation state that does not redraw and/or reproduce the capitalist social division of labour. Such a non-capitalist and transformative state will reconcile intellectual and manual labour and will eliminate private ownership of knowledge and exploitative relations of innovative production. However, all these prospects are theoretical. The key set of questions that we should be asking is rather practical. Namely, is a non-capitalist innovation state feasible in the twenty-first century? If so, does a non-capitalist innovation state have to be necessarily a socialist one in the Poulantzian sense of the term (i.e. democratic socialism)? Is there a pluralism of new alternatives from the bottom up, including some going beyond Poulantzas’ democratic socialism (i.e. his theory of the democratic socialist state)?

Let me attempt to answer these questions by stating the following: Poulantzas’ theory is crucial for understanding the state as a social relation that reflects the division of labour within society. This understanding is missing from contemporary innovation theories of the entrepreneurial state, leading them to reach superficial conclusions about changing capitalism from the top-down. However, despite its importance for the innovation state, Poulantzas’ theory also faces limitations. First, as many scholars (Bruff 2012; Mueller 2019) have pointed out, it remains structuralist Marxist and unable to account fully for the importance of agency in social change. Second, this theory remains somewhat abstract and unable to fully account for empirical developments towards multiple alternatives to the capitalist state of innovation. With exception his *Fascism and Democracy* (1974) (that applies the conceptual and epistemological formulations of his *Political Power and Social Classes* (1973)), Poulantzas maintains a rather formalistic approach to theorising the interplay between state, institutions, and political struggles. This implies difficulty of grasping the plurality and the role of social movements and campaigns which are not rooted in class struggle. For Poulantzas, political mobilisation to transform state structures and to alter policies can only be explained in terms of class struggle. Movements and campaigns which are not directly rooted in class struggle seem to be less effective in challenging and capturing the state. Often, these movements and campaigns are not viewed as genuine alliances. In Poulantzas’s theory, as Jessop (1982: 165) argues, ‘… state power … corresponds unequivocally to the interests of the power bloc and … the working class cannot advance its fundamental interests … and/or secure its own hegemony through the capitalist state …. Yet, while the dominated classes cannot establish their own state power simply through the capture of the existing state apparatus and must develop their own class unity in and through the struggle for a new form of state, they are present in the capitalist state in a disunified, fragmented manner and can advance their particular isolated ‘economic-corporate’ interests through this state to the extent that such advances also sustain bourgeois hegemony’. Poulantzas’ emphasis on the importance of working classes unity takes us back to Gramsci who understood the capitalist state to play crucial role in reconciling particular interests of different fractions within working classes through projects and hegemonic visions in an ‘illusionary’ notion of general interest (Jessop 2016: 215). However, this seems to underplay the importance of radical yet fragmented movements and campaigns in challenging the capitalist state and offering alternatives. As Jackson and Lamb (2021: 6) point out, such movements and campaigns are part of civil society and range from ‘… concerned consumer shopping for fair trade goods in the supermarket, the car-free families showing how we can cut carbon emissions, volunteers helping settle refugees, and people protesting against local fracking, to entire grassroots movements of huge NGOs whose operations span the globe’.

Given the crisis of contemporary capitalism and the inability of this system of political economy to address challenges such as climate change and deep socio-economic inequalities, various such movements world-wide point towards public actions and campaigns which aim at changing the social division of labour and promoting transition to an innovation state that takes on board the interests of everyone (i.e. innovators, regulators and publics). Indeed, so much social and political change has come about only because civil society has imagined and felt the responsibility to act for better world (Jackson and Lamb 2021). Quite often, public actions and campaigns are bottom-up mobilisations against innovation injustice (Papaioannou 2018). They are motivated by what Young (2011) calls ‘personal responsibility’ for justice in innovation systems and related structures. According to her ‘what it means to be responsible is for a person to maintain control over his or her actions and their consequences and to make sure that they and only bear their costs’ (Young 2011: 23). The direction of innovation can only change from the bottom-up when individuals realise that the capitalist division of labour fails to deliver innovations and values for everyone and thereby feel responsible for taking political action (Arvidsson 2020). This bottom-up change is by definition conflictual. Political action aims to undermine the coherence of capitalist state apparatus and thereby prevent it from reproducing the social division of labour.

It is this division of labour that lies at the core of the industrial skeleton of the global economy too. Take for example the area of global health. As Srinivas (2021) argues, industries world-wide are too distant from health systems, health governance and related policy design. According to her, their ‘… business model is driven by select, profitable, slivers of industrial activity where thousand cuts have undermined public goods for healthcare …’ (ibid). This implies that we need a non-capitalist innovation state able to reconcile industrial and health structures for the public good. However, such reconciliation will not just happen through institutions, norms, standards and regulations. It will happen through a transition to an innovation state that has abolished social divisions between manual and intellectual labour and/or between private owners of knowledge and public users of knowledge. These sorts of divisions are clearly reflected in industrial separations between manufacturing and delivery and between the public and the private. However, as Srinivas (2021) stresses, success stories of addressing challenges such as COVID-19 indicate the importance of closing such separations and allowing a range of stakeholders to play different non-hierarchical roles ranging from administrating lockdowns to delivering vaccines. Equal access to medicines, vaccines, and medical devices, including diagnostics, presupposes that everyone’s interest is taken on board. There should be no exclusions driven by social and economic divisions.

The capitalist innovation state, especially during the historical dominance of neo-liberalism, has justified exclusion on the grounds of individual freedom to maximise exchange-value of innovation through unregulated globalising markets. In contrast, a non-capitalist innovation state can justify inclusion on the grounds of collective freedom to maximise use-value of innovation. Given the crisis of neo-liberal globalisation, a non-capitalist innovation state can regain power and impose constraints on global capital’s interests in exclusive innovation and development. Instead of exclusive technologies, it can promote innovations from the bottom up that have more use-value than exchange-value and satisfy basic human needs. From India to Peru, there have been numerous bottom-up initiatives for inclusive innovations (either grassroots or frugal) which have use-value simply because they address basic human needs (Kothari 2021).

In their book *From Anger to Action*, Jackson and Lamb (2021) provide an extensive account of such initiatives which are no longer wishful thinking but rather build up bottom-up alternatives, meeting real human needs. For example, according to them (ibid: 132), ‘Web-based, non-profit initiatives are proliferating, based on citizens sharing and collaborating across borders for the common good – whether creating high-quality open-source software from Linux operating system or Wikipedia’s extraordinary 46 million articles in 300 languages accessed by 1.4 billion unique devices every month’. Subverting and appropriating technologies for better alternatives to capitalist innovation state and for better futures is what Smith and Fressoli (2021) call post-automation. The latter is, in essence, a bottom-up set of capabilities which open up sociotechnical alternatives to capitalist accumulation and the exploitative division of labour. These capabilities are already important for social movements which promote algorithmic justice, data commons, etc. Martell (2023) notes that people around the world already practice alternatives, including innovative digital alternatives such as Mastodon (which has attracted an exodus from Twitter) and democratically owned technologies. Precondition for these alternatives to scale up is institutional change that does not reproduce the exploitative division of labour.

In a recent article, Smith and Stirling (2018: 66–67) also list a number of other innovations: ‘Wind energy, community supported agriculture, social technologies, car clubs, free software, open hardware, repair cafes, participatory design, agro-ecology, eco-housing, recycling, shared machine shops rainwater harvesting, complementary currencies, credit unions, socially useful production, seed swapping, community energy cooperatives, garden sharing, community forestry, green spaces and many, many other ideas and practices for sustainable development began in innovative grassroots activity’. The common denominator of all these initiatives is the absence of a social division between intellectual and manual labour. As a result of it, there are no hierarchical relations of domination and oppression in the process of generation and application of new knowledge. Instead, there is a new commons generated in the form of ‘general intellect’ (Arvidsson 2020: 25). According to Smith and Stirling (ibid: 69), ‘People bring different forms of expertise and experience into collective behaviour. It can be technical knowledge built up through one’s job or professional training, such as accounting and doing books for the initiative or some engineering knowledge. Or it can be vital expert knowledge dynamics in the neighbourhood and using one standing or contacts to bring people on board and earn legitimacy’.

Absence of hierarchical relations creates the potential for more democratic innovation state that can politically direct technologies and services towards social justice. It is worth pointing out here that a non-capitalist innovation state is not necessarily a socialist state. This is not because a socialist state cannot be innovative but because democratic socialism can be considered to be just one alternative to capitalism. Although this alternative has been theorised in both the global North and global South through the Marxian and neo-Marxist critiques of the capitalist political economy as a transitional stage towards the teleology of a classless society, attempts for its implementation have been problematical in several respects (e.g. democratic engagement) and historically inconsistent with the theory. In the twenty-first century, there may be a plurality of alternatives (including democratic socialist, pluralist socialist, post-socialist, post-colonial, and post-development ones) emerging through public action and social movements especially in the global South. Innovation and political theory should explore this plurality of alternatives further to guide innovation and political practice. It should, for example, explore whether a non-capitalist innovation state might not have to focus on economic growth or to organise centrally knowledge and production activities for the purpose of just innovation. In addition, a non-capitalist innovation state might be guided by human rights and therefore be able to politically direct innovation towards satisfying needs of equal members of society.

It might be argued that a non-capitalist innovation state that is democratic might in theory be even more appropriate for promoting a conception of equality in terms of the relations between individuals in society than a democratic socialist state. Comparing to the latter that conceives equality in terms of distribution of resources, the former can focus on establishing a society of equals through innovation. In the absence of any social division of labour (including racial and gendered forms), this implies reduction of hierarchy, oppression, discrimination, domination, and so forth through innovations such as ICTs (e.g. by recording live cases of oppression and discrimination and addressing them immediately) and new life sciences (e.g. by reducing genetic disorders). A non-capitalist innovation state might even be more pragmatic than a socialist innovation state in the sense that the former’s approach to justice is driven by inequalities on the ground whereas the latter’s approach is driven by an ideal theory of principles which would create a society of equals. A more pragmatic non-capitalist innovation state also implies the acceptance that not all relational inequalities will be eliminated. There may be non-capitalist forms of oppression, e.g. patriarchy deeply embedded in culture, religion, and history of some societies. A non-ideal theory of relational justice should carefully study and understand the nature of such forms of oppression in order to find ways towards just outcomes.

Finally, it can be also proposed that a non-capitalist innovation state can even allow many different ‘societies of equals’ at the same time, depending on what kind of precise relations people accept as justified in their societies. As Wolff (2015: 15) put it, what these societies ‘… have in common is that they avoid various objectionable social relations. These objectionable social relations will fall into at least two groups. One picks out a range of asymmetrical relations, as set out above (oppression, exploitation, etc.) and hence identifies various forms of inequality. The other concerns relations of alienation or estrangement. This latter group picks out the idea that a society of equals is, after all, a *society*, and therefore, should have a certain fabric of mutual connectedness’. In theory, a non-capitalist innovation state can politically direct new technologies to identify and remedy both groups of objectionable social relations. In this sense, it can enable holistic change towards a democratic society of equals.

## Conclusion

This paper has sought to address the question of what kind of innovation state matters for social justice. It has done so by revisiting Poulantzas’ theory of the state and going beyond it to consider alternatives to capitalist innovation state from the bottom up. Theorists of the state of innovation have not paid attention to the ideas of Poulantzas. This lacuna in scholarly attention has implications for failing to understand the state of innovation as a social relation that reflects the capitalist division of labour. The argument put forward has been that theorists of innovation should learn from Poulantzas and should re-think the state not in terms of combining Keynesian and Schumpeterian accounts but in terms of changing the fundamental division of labour within capitalism. It is this social division that underpins the state and diverts innovation towards exploitation and injustice. Unless it is changed or eliminated from the bottom up and through public action of politically responsible individuals, the social division of labour within capitalism will carry on producing exclusions from innovation and promote private ownership of dominant social classes of technologies. This paper has discussed the possibility of a non-capitalist innovation state, recognising the shortcomings of Poulantzas’ theory. These shortcomings have to do with the formalistic and structuralist nature of this theory that does not fully account for the plurality of bottom-up alternatives to the capitalist state. Yet, such alternatives can only be the fruit of public action and campaigning for closing social divisions in innovative production. Instead of these divisions, a non-capitalist innovation state can focus on knowledge sharing for products and processes which have use-value. It can do so by restricting or even eliminating IPR regimes and promoting collective action for the common good.

## Footnotes

1 The notion of market fundamentalism has been used by scholars such as Block (2013) to describe the 1980s’ neo-liberal turn to free market competition and limited government. These institutions have been presented as natural, overlooking the importance of legal and institutional practices which very much shape them. For example, as Davis and Klare (2010: 409) remind us ‘…the so-called ‘freedom of contract’ consists of negotiation conducted and agreement made within governmentally structured framework of mutual pressure and coercion’

2 This point was made by one of REPE editors. I would like to thank her for the contribution

3 It is important to notice here that Amsden (1989), long-time before Mazzucato and Block developed their positions on innovation state, argued that the state in late industrialised countries of East Asia played the role of entrepreneur in economy, disciplining capital to be invested in specific industries for long-term development

4 This point was made by one of REPE reviewer. I would like to thank him/her for the contribution
